# A House Epidemic of Syphilis

**Published:** 1899-03

**Authors:** William S. Gottheil


### A House Epidemic of Syphilis.

BY WILLIAM S. GOTTHEIL, M. D.

Thanks to a better knowledge of the dangers and modes of transmission of syphilis, and to superior habits of cleanliness, epidemics of the disease are rare in America; yet they occur among the lower classes of our population with greater frequency than is generally supposed. In the *New York Medical Journal* of March 26th, the writer records one in which the disease was introduced into the family, according to the history, by vaccination, and in which every member of the family of eight was ultimately infected. The first case was a child of 2 years; then the mother, aged 34; then two girls, aged 9 and 14 respectively; then a boy of 4; then a girl of 7; and then a nurseling, aged six months. The father escaped until the last; but late in the spring he came to the clinic with a characteristic eruption, alopecia, etc. The cases were all severe; there were several irites; all had obstinate and some very extensive mucous patches; and the 2 year old child had a syphilitic pneumonia. The site of inoculation was discoverable in two cases only, probably on account of the lateness and irregularity with which the patients were brought to the clinic. In the mother it was upon the center of the cheek, and in one girl it was upon the eyelid. The family was very poor, living in one room, and their habits were very uncleanly.

